# Thioredoxin mitigates H_2_O_2_‐induced inhibition of myogenic differentiation of rat bone marrow mesenchymal stem cells by enhancing AKT activation

**DOI:** 10.1002/2211-5463.12835

**Published:** 2020-04-02

**Authors:** Meiling Liu, Xianglu Li, Changlin Zhou, Manfeng Wang, Hongzhi Wang, Haifeng Ding, Luyang Cheng, Lu Gan, Xiaowei Wu, Zhimin Du

**Affiliations:** ^1^ Department of Geriatrics The Second Affiliated Hospital of Harbin Medical University China; ^2^ Department of Orthopedics The Second Affiliated Hospital of Harbin Medical University China; ^3^ Institute of Clinical Pharmacology The Second Affiliated Hospital of Harbin Medical University China; ^4^ State Key Laboratory of Quality Research in Chinese Medicines Macau University of Science and Technology Harbin China

**Keywords:** Akt, bone marrow mesenchymal stem cells, human recombinant thioredoxin, hydrogen peroxide, myogenic differentiation, thioredoxin

## Abstract

Thioredoxin (Trx) is a hydrogen acceptor of ribonucleotide reductase and a regulator of some enzymes and receptors. It has been previously shown that significantly elevated levels of Trx expression are associated with the osteogenic differentiation of bone marrow mesenchymal stem cells (BMSCs), but it is not clear how Trx regulates the effects of hydrogen peroxide (H_2_O_2_) on myogenic differentiation of BMSCs. Here, we report that rat BMSCs treated with a high dose (150 µm) of H_2_O_2_ exhibited a significant reduction in viability, cell cycling, and superoxide dismutase and glutathione peroxidase levels, and an increase in reactive oxygen species and malondialdehyde levels, which was accompanied by reductions in protein kinase B activation and forkhead Box O1, myogenic differentiation 1 and myogenin expression during myogenic differentiation. Furthermore, treatment with recombinant human Trx significantly mitigated the effects of H_2_O_2_ on the myogenic differentiation of BMSCs, and this was abrogated by cotreatment with wortmannin [a specific phosphatidylinositol 3‐kinase inhibitor]. In summary, our results suggest that treatment with recombinant human Trx mitigates H_2_O_2_‐induced oxidative stress and may promote myogenic differentiation of rat BMSCs by enhancing phosphatidylinositol 3‐kinase/protein kinase B/forkhead Box O1 signaling.

## Graphical abstract

Hydrogen peroxide can cause significant peroxidative damage to and inhibit the proliferation and myogenic differentiation of bone marrow mesenchymal stem cells (BMSCs). In this study, we found that recombinant human Trx significantly mitigated hydrogen peroxide‐induced oxidative stress and may promote myogenic differentiation of rat BMSCs by enhancing phosphatidylinositol 3‐kinase/protein kinase B/forkhead Box O1 signaling, which may provide an experimental basis for the BMSC‐based treatment of sarcopenia.

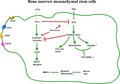

**Abbreviations**
AKT: protein kinase B
BMSC: bone marrow mesenchymal stem cell
DMEM: Dulbecco’s modified Eagle’s medium
FoxO1: forkhead Box O1
GSH‐PX: glutathione peroxidase
H_2_O_2_: hydrogen peroxide
MDA: malondialdehyde
MTT: 3‐(4,5‐dimethylthiazol‐2‐yl)‐2,5‐diphenyl‐2‐H‐tetrazolium bromide
MyoD1: myogenic differentiation 1
OM: third passage of BMSCs was cultured in myogenic differentiation medium
P3: third passage of BMSCs
PI3K: phosphatidylinositol 3‐kinase
rhTrx: recombinant human thioredoxin
ROS: reactive oxygen species
SEM: standard error of the mean
SOD: superoxide dismutase
TBST: Tris‐buffered saline Tween 20
TRX: thioredoxin
Wor: wortmannin

Bone marrow mesenchymal stem cells (BMSCs) have multiple differentiation potentials, which are dependent on the cell environment. Previous studies have shown that BMSCs can differentiate into muscle cells in a given condition, which is positively regulated by muscular transcription factors myogenic differentiation 1 (MyoD1) and myogenin [1, 2]. During the process of aging, aberrant oxidative stress can result in the peroxidation of muscular tissues, leading to their atrophy [3]. Furthermore, aberrant oxidative stress can induce high levels of reactive oxygen species (ROS) production and inhibit the proliferation and osteogenic differentiation of BMSCs [4]. However, it remains unclear how ROS affects the myogenic differentiation of BMSCs.

Thioredoxin (Trx) is an evolutionarily and highly conserved protein that is expressed widely in prokaryotic and eukaryotic cells. Furthermore, Trx is a hydrogen acceptor of ribonucleotide reductase and a regulator of some enzymes and receptors. Previous studies have shown that recombinant human Trx (rhTrx) can protect cardiomyocytes from hypoxic injury through its antioxidant and anti‐inflammatory activity [5]. Treatment with rhTrx inhibits foam cell formation [6] and reverses aging‐related hypertension [7]. A previous study indicated that significantly elevated levels of Trx expression are associated with the osteogenic differentiation of BMSCs, suggesting that Trx may positively promote the osteogenic differentiation of BMSCs [8]. However, there is no information on whether Trx can modulate the myogenic differentiation of BMSCs and how Trx regulates the hydrogen peroxide (H_2_O_2_)‐modulated myogenic differentiation of BMSCs and their myogenic transcription factor expression.

This study used rat BMSCs as a model to test the effect of rhTrx on H_2_O_2_‐modulated myogenic differentiation and explore the potential mechanisms. Furthermore, this study aimed to develop an effective method for enhancing BMSC myogenic differentiation, which may provide an experimental basis for the BMSC‐based treatment of sarcopenia.

## Materials and methods

### Materials and reagents

Male Sprague Dawley rats were obtained from the Animal Experimental Center of The Second Affiliated Hospital of Harbin Medical University. The other specific reagents included Dulbecco’s modified Eagle’s medium (DMEM)/F12 medium (HyClone, Logan, UT, USA); FBS (Biological Industries, Beit Haemek, Israel); horse serum, SDS/PAGE gel preparation and ROS test kits (Beyotime Biotechnology, Shanghai, China); basic fibroblast growth factor (bFGF; PeproTech, Rocky Hill, NJ, USA); superoxide dismutase (SOD) and malondialdehyde (MDA) test kits and glutathione peroxidase (GSH‐PX; Nanjing Institute of Bioengineering, Nanjing, China); cell cycle detection and supersensitive ECL luminescent kits (HaiGene, Harbin, China); monoclonal antibodies against CD44, CD90, CD29, CD105, CD31 or CD34 and goat anti‐(rabbit IgG‐Cy3) serum (Absin, Shanghai, China); rhTrx (R&D Systems, Minneapolis, MN, USA); antibodies against MyoD1 and myogenin (Abcam, Cambridge, UK); antibodies against protein kinase B (AKT), phospho‐AKT, forkhead Box O1 (FoxO1) and wortmannin (Wor; Cell Signaling Technology, Danvers, MA, USA); goat anti‐(mouse IgG‐fluorescein 5‐isothiocyanate) serum and goat anti‐[mouse (H + L)/horseradish peroxidase] serum (Zhongsu Jinqiao, Beijing, China); and dexamethasone (Solarbio, Beijing, China).

The myogenic differentiation induction medium was DMEM/Ham’s F12 containing 2% horse serum, 1% glutamine, 1 ng·mL^−1^ basic fibroblast growth factor and 0.4 µg·mL^−1^ dexamethasone without antibiotics [9].

### Materials and methods

#### Cell culture

Male Sprague Dawley rats (3–4 weeks old) were sacrificed, and the periosteum and muscle tissues were dissected. Their bone medullary cavity was repeatedly washed with DMEM/F12 medium containing 10% FBS, and the flow‐through cell suspension was centrifuged. The pelleted cells were cultured in the differentiation medium at 37 °C with 5% CO_2_ for 48 h and exposed to fresh medium. One day later, when the cells reached 80% confluence, these cells were passaged with fresh medium every 3 days up to the third generation for the experiments. The cell culture medium was changed at 48 h postpassage. The animal experiments were approved by the Ethics Committee of The Second Affiliated Hospital of Harbin Medical University.

#### Immunofluorescent analysis of BMSCs

BMSCs at the third passage were fixed with 4% paraformaldehyde for 15 min and blocked with 5% BSA in Tris‐buffered saline Tween 20 (TBST). These cells were probed with primary antibodies (1 : 100) against CD44, CD90, CD29, CD105, CD31 and CD34 overnight at 4 °C, and bound antibodies were detected with the Cy3‐conjugated goat anti‐(rabbit IgG) serum (1 : 400) and fluorescein 5‐isothiocyanate‐conjugated goat anti‐(mouse IgG) serum (1 : 100), followed by nuclear staining with 4′,6‐diamidino‐2‐phenylindole. The negative control cells were treated with PBS to replace the primary antibody, and the fluorescent signals of cells were observed under a fluorescent microscope (Nikon Eclipse Ti, Nikon, Japan).

#### Grouping and treatment

BMSCs were cultured in DMEM/F12 or treated with rhTrx and/or 150 µm H_2_O_2_ or together with 0.5 μm Wor in the differentiation medium for 24 h (Table 1).

**Table 1:** The experimental groups and treatments.

Group	Medium	Treatment
P3	DMEM/F12	No
OM	Myogenic differentiation medium	No
OM + rhTrx	Myogenic differentiation medium	2 μg·L^−1^ rhTrx
OM + H_2_O_2_	Myogenic differentiation medium	150 µm H_2_O_2_
OM + rhTrx + H_2_O_2_	Myogenic differentiation medium	2 μg·L^−1^ rhTrx + 150 µm H_2_O_2_
OM + rhTrx + H_2_O_2_ + Wor	Myogenic differentiation medium	2 μg·L^−1^ rhTrx + 150 µm H_2_O_2_ + 0.5 μm Wor

After being washed with the culture medium, the cells were continually cultured in the same medium for 48 h to detect the oxidative stress, cell cycle and protein expression. The remaining cells were continually cultured for 14 days to detect the MyoD1, myogenin and caspase‐3 expression.

#### Cell viability

To evaluate the potential cytotoxicity of H_2_O_2_, we cultured BMSCs (1 × 10^4^ cells per well) in 96‐well plates and treated them in triplicate with different doses (50, 100, 150, 200 or 400 μm) of H_2_O_2_ for 24 h. During the last 4 h of culture, the cells were exposed to 20 μL (5 g·L^−1^) 3‐(4,5‐dimethylthiazol‐2‐yl)‐2,5‐diphenyl‐2‐H‐tetrazolium bromide (MTT) solution, the resulting formazan in the individual wells was dissolved in 100 μL DMSO, and the absorbance was measured at 490 nm using a microplate reader. Further experiments were performed by treating cells in triplicate with 150 μm H_2_O_2_ and/or rhTrx and Wor for 24 h, and changing to the differentiation medium for 48 h to determine the cell viability in each group by MTT assay.

#### Determination of oxidative stress

BMSCs (1 × 10^4^ cells per well) were cultured in 96‐well plates and treated in triplicate with the reagents described earlier. The levels of intracellular ROS, MDA and GSH‐PX, and SOD activity in individual groups of cells were determined, according to the manufacturer’s instructions. The experimental and control cells were simultaneously tested. In brief, the levels of intracellular ROS were determined using the DFCH‐DA solution provided in the kit and measured using a fluorescence microplate reader at 488 nm. The levels of intracellular GSH‐PX were determined using a microplate reader at 412 nm. The levels of intracellular MDA in the individual groups of cell homogenates were quantified using the provided specific reagents in a microplate reader at 532 nm. The level of SOD activity of cells in the individual wells of cells was examined by SOD inhibition assay.

#### Analysis of cell cycling

Individual groups of cells were harvested, washed twice with ice‐cold PBS and fixed in 75% EtOH at 4 °C overnight. The cells were stained with propidium iodide staining buffer, which contained 1 mg·mL^−1^ RNase A, for 30 min in the dark. After being washed gently with ice‐cold PBS, the cells were analyzed by flow cytometry. The data were analyzed using the modfit software (Verity Software House, Topsham, ME, USA).

#### Immunofluorescent analysis of MyoD1 and myogenin expression

After the 14‐day differentiation, the individual groups of cells were fixed with 4% paraformaldehyde and permeabilized with 1% Triton X‐100, followed by blocking with 2% BSA in TBST. The cells were probed with primary antibodies (1 : 100) against MyoD1 or myogenin at 4 °C overnight. After being washed with TBST, the bound antibodies were detected using the fluorescent secondary antibody (1 : 200) for 1 h in the dark and nuclear stained with 4′,6‐diamidino‐2‐phenylindole. The fluorescent signals were photographed using a fluorescence microscope and analyzed by the imagej software (Rawak Software, Inc. Stuttgart, Germany).

#### Western blot

These individual groups of cells were harvested, lysed in immunoprecipitation assay buffer and centrifuged. The protein concentrations in these individual samples were determined by bicinchoninic acid assay. The cell lysates (20 µg per lane) were separated by SDS/PAGE on 10% gels and transferred onto nitrocellulose membranes. The membranes were blocked with 5% skimmed dry milk in TBST, probed with the indicated primary antibody (1 : 1000) at 4 °C overnight and detected with the horseradish peroxidase‐conjugated secondary antibody. After being washed with TBST, the immunocomplex was visualized using the enhanced chemiluminescent reagents and analyzed by imagej software.

### Statistical analysis

The data are expressed as mean ± standard error of the mean (SEM) because all data were normally distributed. The difference among groups was evaluated by one‐way ANOVA and *post hoc* Bonferroni test, and the difference between groups was analyzed by Dunnett’s T3 and least significant difference test using the spss 24.0 software (International Business Machines Corporation (IBM), Armonk, NY, USA). A two‐tailed *P*‐value <0.05 was considered statistically significant.

## Results

### Isolation and characterization of rat BMSCs

**Fig. 1:**
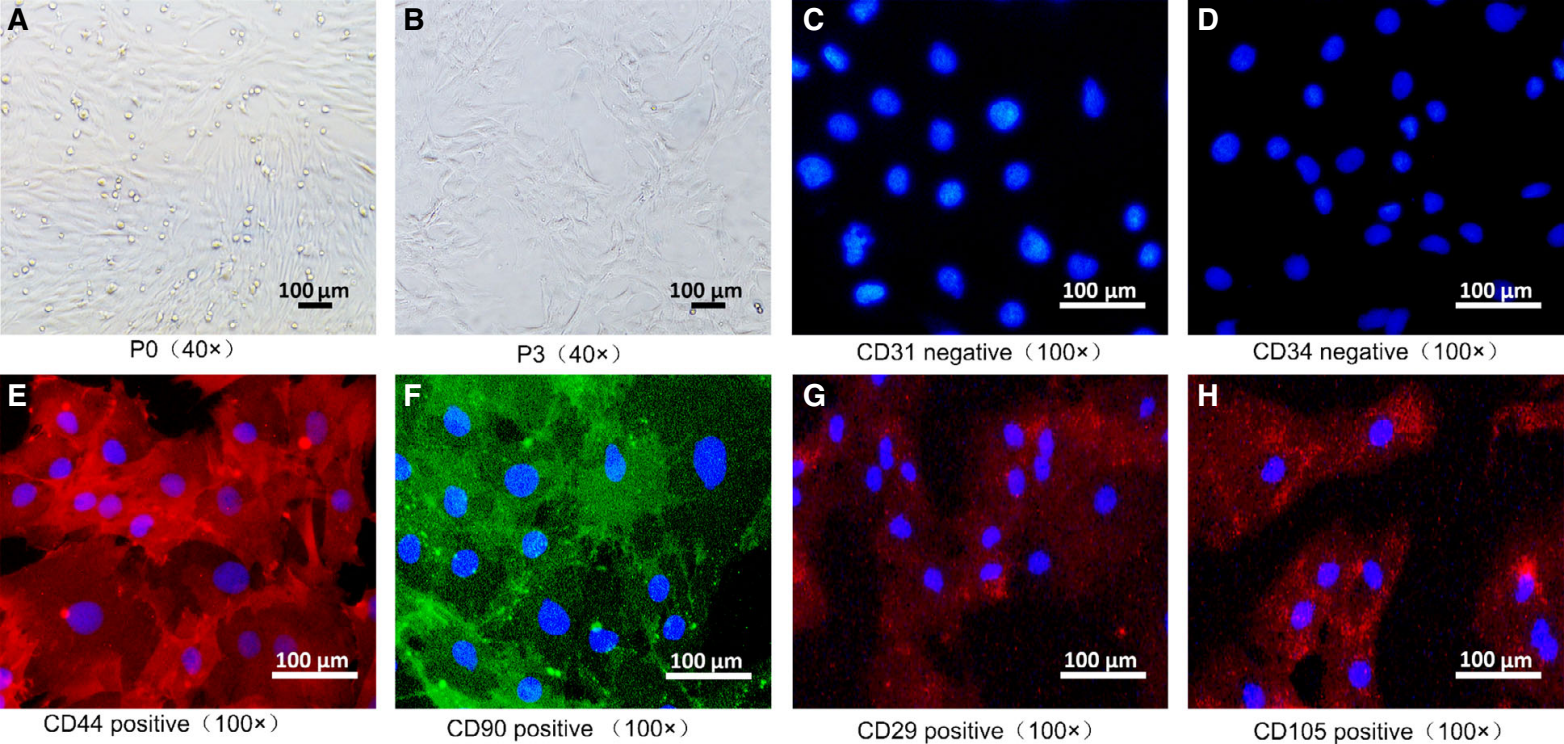
Characterization of BMSCs. Rat BMSCs were isolated and cultured for 3 days (P0), or induced for myogenic differentiation for up to three passages (P3) and photographed. The expression of CD44, CD90, CD29, CD105, CD31 and CD34 in the differentiated cells was characterized by indirect immunofluorescence assay using primary monoclonal antibodies against the indicated antigens and fluorescent secondary antibodies. Negative control cells were treated with PBS to replace the primary antibody. The data are representative images with the indicated magnifications obtained from three separate experiments. (A) P0 BMSCs cultured for 72 h. (B) P3 BMSCs. (C) Negative CD31 expression in BMSCs. (D) Negative CD34 expression in BMSCs. (E) Positive for CD44 expression in P3 BMSCs. (F) Positive for CD90 expression in P3 BMSCs. (G) Positive for CD29 expression in P3 BMSCs. (H) Positive for CD105 expression in P3 BMSCs. Scale bars: 100 µm.

Primary rat BMSCs were isolated and cultured for 72 h. The adherent cells presented a fibroblast‐like morphology (Fig. 1A). After the induction of myogenic differentiation for three passages, these cells had a uniform morphology, with a spiral and fencelike arrangement (Fig. 1B). Immunofluorescent staining indicated that these cells were negative for CD31 and CD34 expression (Fig. 1C,D), but positive for CD44, CD90, CD29 and CD105 expression (Fig. 1E–H). Hence these isolated cells had the phenotype characteristics of BMSCs.

### Treatment with rhTrx mitigates the cytotoxicity of H_2_O_2_ in primarily cultured rat BMSCs

**Fig. 2:**
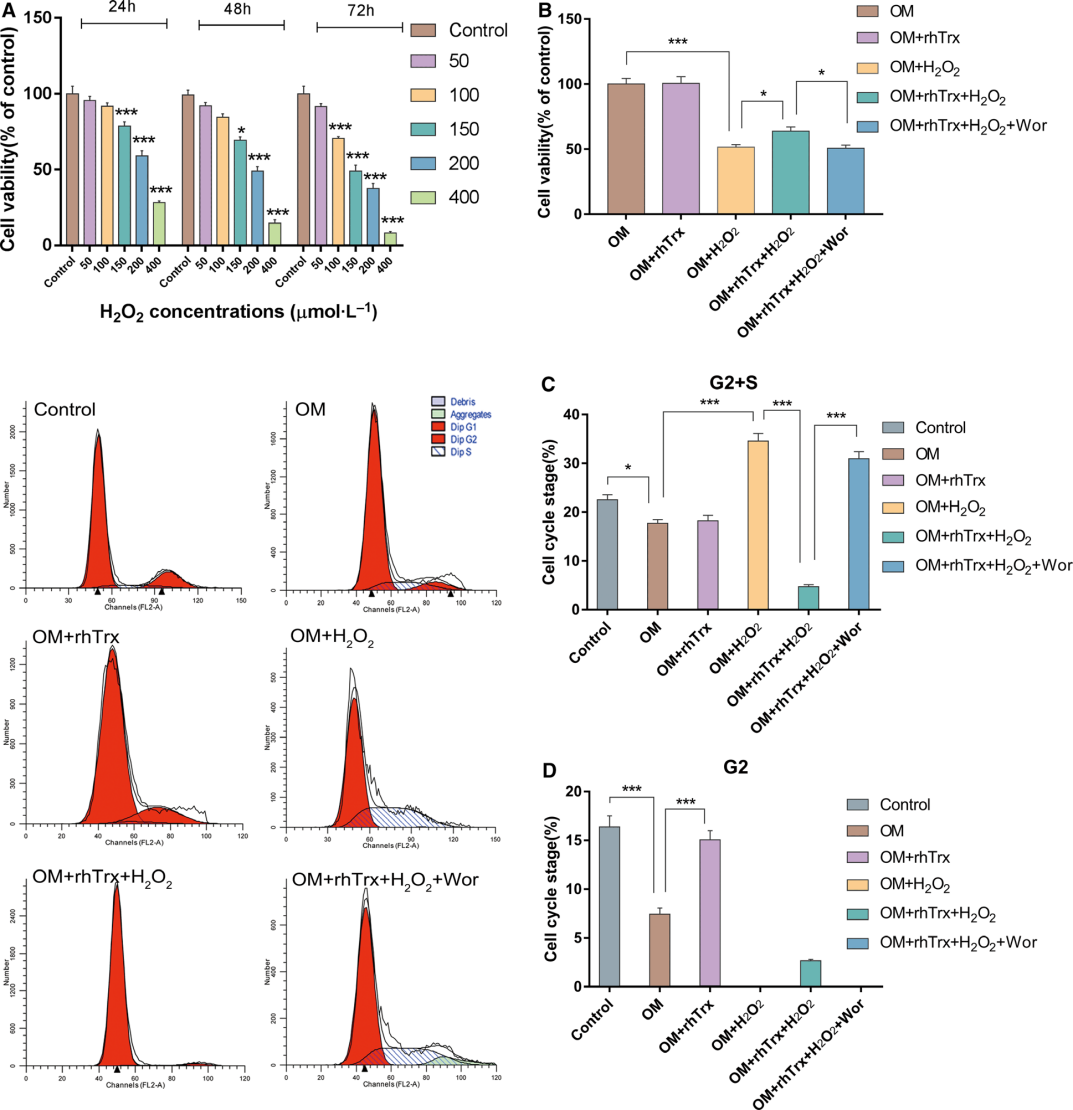
The treatment with rhTrx mitigates the cytotoxicity of H_2_O_2_ in primarily cultured rat BMSCs. Rat BMSCs were treated in triplicate with the vehicle control, or with 50–400 µm H_2_O_2_ for 24–72 h, and the viability was determined by MTT assay. Subsequently, these BMSCs were treated with the indicated reagents, and the viability of these different groups of cells was determined by MTT assay. Then the cell cycling in these different groups of cells was analyzed by flow cytometry. The data are representative of the flow cytometry histograms or are expressed as the mean ± SEM of each group of cells obtained from three separate experiments. (A) The dose‐dependent effect of H_2_O_2_ on the viability of BMSCs. (B) The treatment of rhTx mitigates the H_2_O_2_‐mediated cytotoxicity against BMSCs. (C, D) The flow cytometry analysis of cell cycling. *P < 0.05; ***P < 0.001.

To determine the role of H_2_O_2_ in regulating the myogenic differentiation of rat BMSCs, we first determined the cytotoxicity of H_2_O_2_ against rat BMSCs *in vitro*. The primarily cultured BMSCs were treated with different doses (50–400 µm) of H_2_O_2_ for 24–72 h, and the cell viability was determined by MTT assay (Fig. 2A). Treatment with 50 µm H_2_O_2_ did not significantly affect the cell viability in the present experimental condition. However, treatment with a higher dose of H_2_O_2_ significantly reduced the cell viability (*P* < 0.05, *P* < 0.001), and the cytotoxic effects of H_2_O_2_ on the viability of rat BMSCs were dose and time dependent. Given that 150 μm H_2_O_2_ for 24 h significantly decreased the viability of BMSCs, this dose was chosen for the subsequent experiments. Treatment with 2 µg·L^−1^ rhTrx alone did not affect the viability of BMSCs, but significantly mitigated the H_2_O_2_‐decreased viability of BMSCs, indicating that rhTrx protected BMSCs from H_2_O_2_‐mediated cytotoxicity (*P* < 0.05; Fig. 2B). The protective effect of rhTrx was abrogated by treatment of Wor (*P* < 0.05).

Cell cycling analysis revealed that culture of BMSCs in the differentiation medium decreased the percentage of cells in the G2+S and G2 phases, whereas the treatment with 150 µm H_2_O_2_ significantly increased the frequency of cells in the G2/S phase, but decreased them in the G2 phase (*P* < 0.001; Fig. 2C,D). Treatment with rhTrx alone did not change the percentages of BMSCs in the G2/S phase, whereas the same treatment significantly increased the frequency of cells in the G2 phase (*P* < 0.01). Furthermore, treatment with rhTrx significantly mitigated the H_2_O_2_‐mediated changes in the frequency of cells in the G2/S and G2 phases, which were abrogated by treatment with Wor (*P* < 0.001). Collectively, these data indicated that rhTrx protected rat BMSCs against H_2_O_2_‐mediated cytotoxicity by enhancing the phosphatidylinositol 3‐kinase (PI3K)/AKT signaling to modulate the cell cycling.

### Treatment with rhTrx inhibits H_2_O_2_‐induced oxidative stress in rat BMSCs

**Fig. 3:**
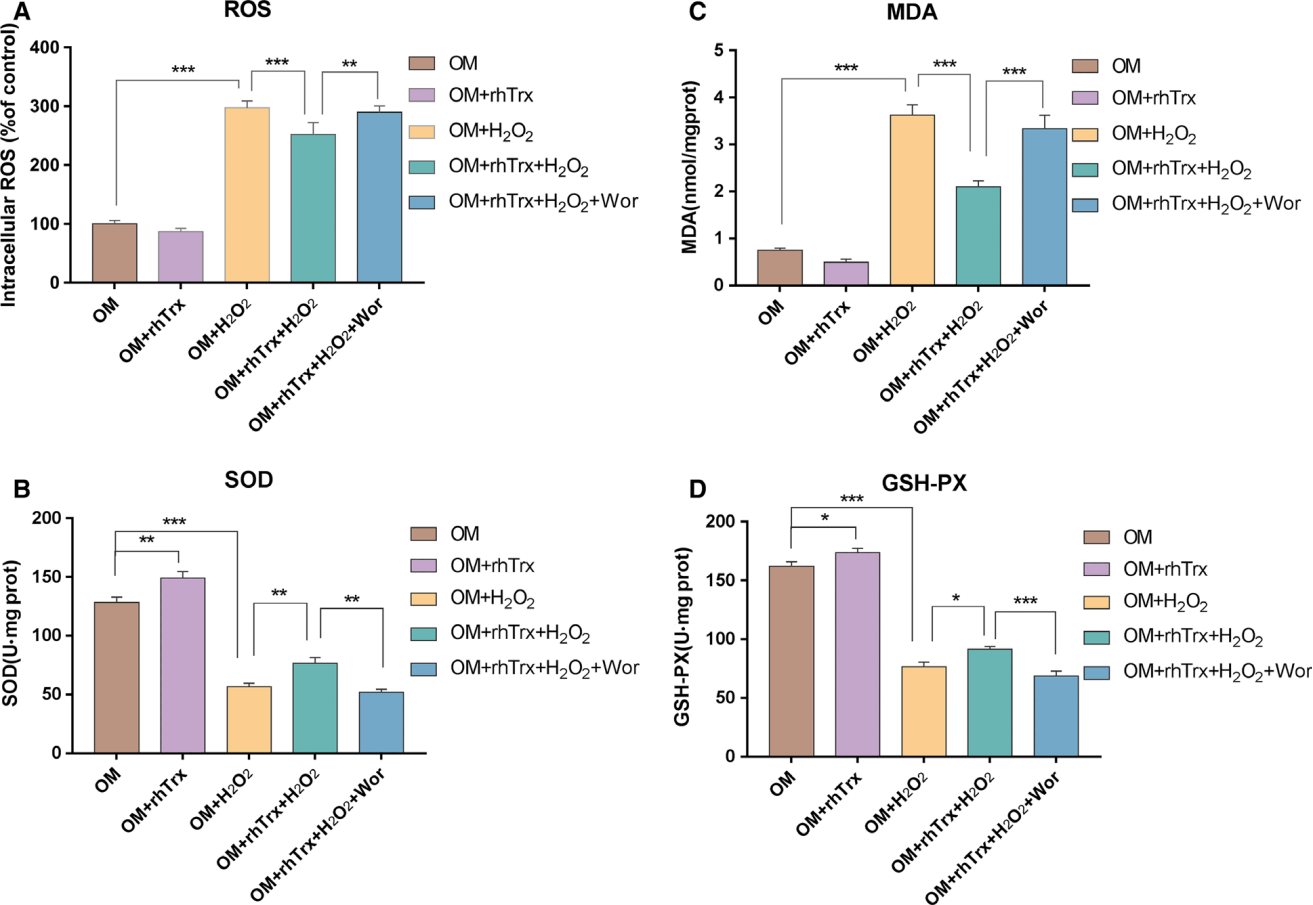
The treatment with rhTrx inhibits the H_2_O_2_‐induced oxidative stress in BMSCs. After treatment with the indicated reagents, the levels of intracellular ROS, MDA and GSH‐PX, and SOD activity in the different groups of cells were measured. The data were expressed as mean ± SEM of each group of cells obtained from three separate experiments. (A) The levels of ROS. (B) The levels of MDA. (C) The levels of SOD activity. (D) The levels of GSH‐PX. *P < 0.05; **P < 0.01; ***P < 0.001.

H_2_O_2_ can induce oxidative stress. To understand the role of rhTrx in mitigating the cytotoxicity of H_2_O_2_, we examined the effect of rhTrx on H_2_O_2_‐induced oxidative stress in BMSCs. Compared with the OM group [third passage of BMSCs (P3) was cultured in myogenic differentiation medium], treatment with rhTrx alone significantly increased the levels of SOD activity and GSH‐PX in BMSCs (*P* < 0.01, Fig. 3C; *P* < 0.05, Fig. 3D). Furthermore, treatment with H_2_O_2_ significantly increased the levels of ROS and MDA, but decreased the SOD and GSH‐PX in BMSCs (*P* < 0.001). In addition, treatment with rhTrx significantly mitigated the levels of H_2_O_2_‐increased ROS and MDA, and H_2_O_2_‐decreased SOD and GSH‐PX in BMSCs (*P* < 0.05, *P* < 0.001; Fig. 3A–D). The modulatory effects of rhTrx on H_2_O_2_‐induced oxidative stress were completely abrogated by the cotreatment with Wor in BMSCs. Hence treatment with rhTrx inhibited the H_2_O_2_‐induced oxidative stress in BMSCs.

### Treatment with rhTrx enhances AKT activation to up‐regulate FoxO1 expression during the myogenic differentiation of rat BMSCs

**Fig. 4:**
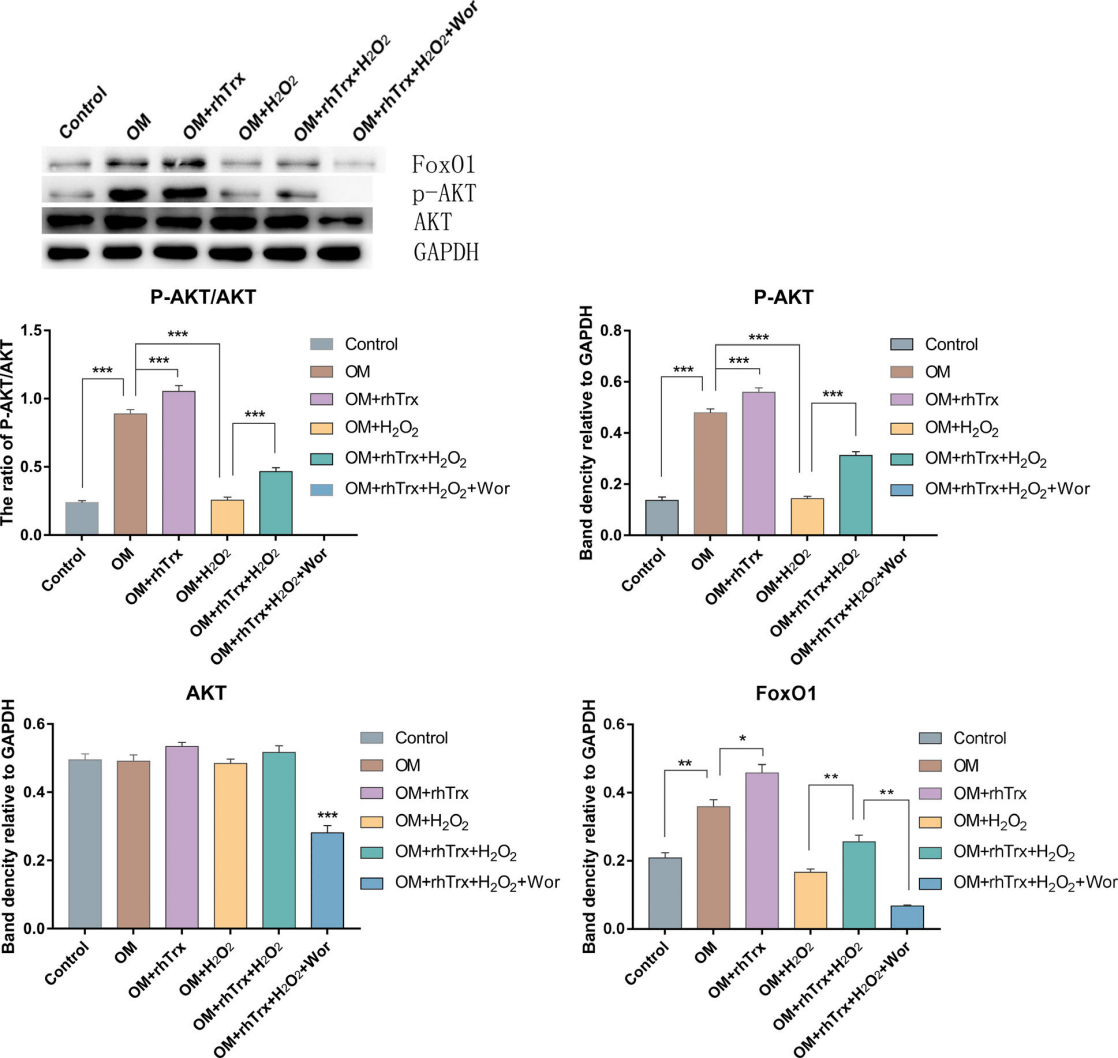
The treatment with rhTrx enhances the AKT activation to up‐regulate FoxO1 expression during the myogenic differentiation of rat BMSCs. The relative levels of AKT expression and phosphorylation, as well as the FoxO1 expression, in the different groups of cells were determined by western blot. The data are the representative images or are expressed as mean ± SEM of each group of cells obtained from three separate experiments. *P < 0.05; **P < 0.01; ***P < 0.001.

Given that treatment with the PI3K inhibitor of Wor abrogates the effect of rhTrx on the H_2_O_2_‐reduced viability of BMSCs, the effect of rhTrx on the AKT activation and downstream FoxO1 expression in BMSCs during the myogenic differentiation was determined by western blot. Wor treatment, but not another treatment, significantly decreased the levels of AKT expression, relative to that in the control BMSCs (Fig. 4). In comparison with that in the control without the induction of myogenic differentiation (P3), the induction of myogenic differentiation significantly increased the relative levels of AKT phosphorylation and FoxO1 expression (*P* < 0.05), and treatment with rhTrx further increased the relative levels of AKT phosphorylation and FoxO1 expression in BMSCs (*P* < 0.05). Furthermore, treatment with H_2_O_2_ significantly decreased AKT phosphorylation and FoxO1 expression, whereas treatment with rhTrx partially rescued the H_2_O_2_‐decreased AKT phosphorylation and FoxO1 expression in BMSCs. Finally, treatment with Wor completely blocked the AKT phosphorylation and further enhanced the H_2_O_2_‐decreased FoxO1 expression in BMSCs, even with the presence of rhTx. Thus, rhTrx partially rescued the H_2_O_2_‐inhibited AKT activation and downstream FoxO1 expression in BMSCs during the myogenic differentiation.

### Treatment with rhTrx mitigates the H_2_O_2_‐inhibited myogenic differentiation of rat BMSCs

**Fig. 5:**
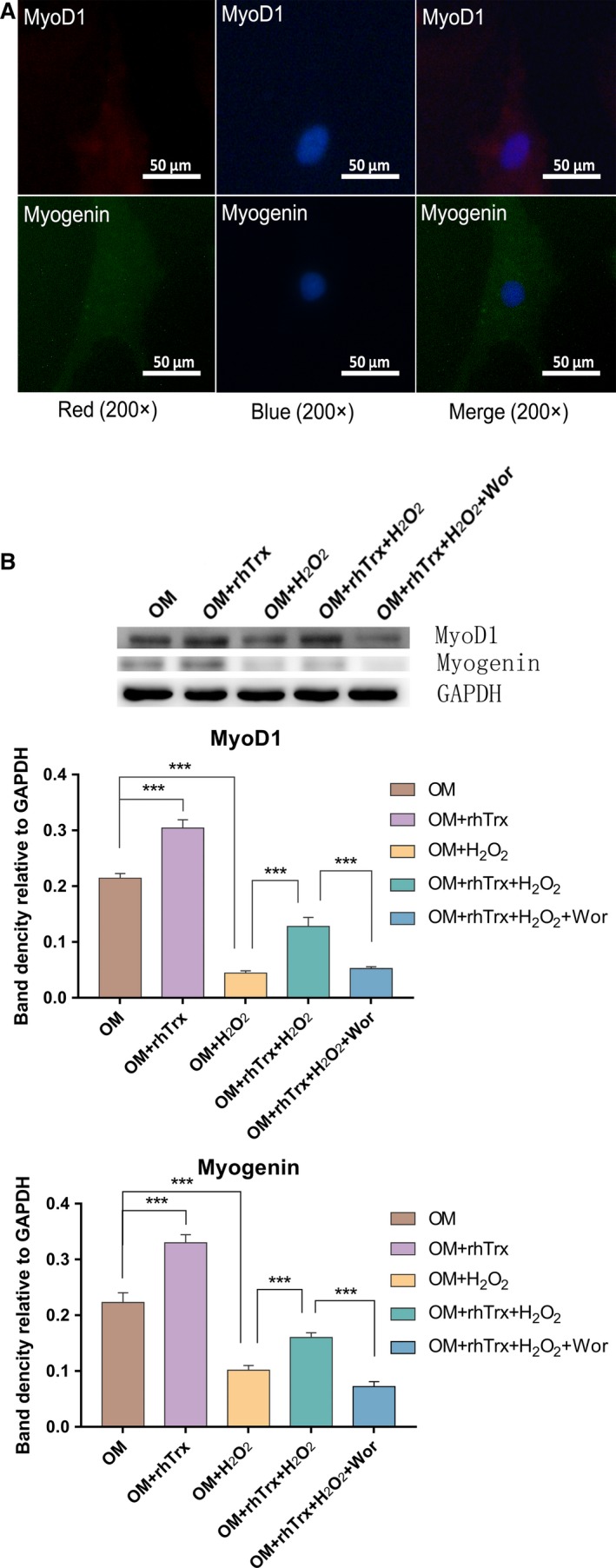
The treatment with rhTrx partially rescues the H_2_O_2_‐inhibited myogenic differentiation in rat BMSCs. The relative levels of MyoD1 and myogenin expression in the different groups of cells were determined by immunofluorescence and western blot. The data are the representative images or are expressed as mean ± SEM of each group of cells obtained from three separate experiments. (A) Immunofluorescence analysis (original magnification: ×200, scale bars: 50 µm). (B) Western blot analysis. ***P < 0.001.

MyoD1 and myogenin are important transcription factors for the myogenic differentiation of BMSCs. Accordingly, the effect of H_2_O_2_ and/or rhTrx on the levels of MyoD1 and myogenin expression in BMSCs after myogenic differentiation for 14 days was examined. Immunofluorescent analysis indicated that MyoD1 and myogenin were positively expressed in BMSCs (Fig. 5A). Compared with the control OM group, treatment with rhTrx significantly increased the relative levels of MyoD1 and myogenin expression (*P* < 0.05), whereas treatment with H_2_O_2_ significantly decreased the levels of MyoD1 and myogenin expression in BMSCs (*P* < 0.01; Fig. 5B). In addition, treatment with rhTrx partially rescued the H_2_O_2_‐decreased MyoD1 and myogenin expression in BMSCs, which were abrogated by Wor treatment (*P* < 0.01 for both). Therefore, treatment with rhTrx significantly mitigated the H_2_O_2_‐inhibited myogenic differentiation in rat BMSCs *in vitro*.

## Discussion

BMSCs are characterized by the expression of CD44, CD90, CD29 and CD105, but not the expression of CD31, CD34 and CD45 [10, 11]. In this study, rat BMSCs were isolated, and BMSCs displayed with a typical morphology and were positive for CD44, CD90, CD29 and CD105 expression, but negative for CD31 and CD34 expression. Because BMSCs can differentiate into multiple types of cells, depending on the differentiation conditions, and have potent immunoinhibitory activity, BMSCs have been tested for treatment of several diseases, including type 1 diabetes, inflammatory bowel disease, rheumatoid arthritis and other diseases [12, 13, 14]. Furthermore, BMSCs have also been tested for intervention of musculoskeletal diseases, which are the main causes of morbidity [15, 16].

Given that oxidative stress can affect the myogenic differentiation of BMSCs and Trx is a potent antioxidant, we investigated the role of rhTrx in regulating the H_2_O_2_‐modulated myogenic differentiation of rat BMSCs *in vitro*. In this study, we found that treatment with a higher dose (150 µm) of H_2_O_2_ significantly reduced the viability of BMSCs in a dose‐ and time‐dependent manner, and this was accompanied by inhibiting cell cycling. Furthermore, treatment with H_2_O_2_ also significantly increased the levels of ROS and MDA, but decreased the SOD activity and GSH‐PX levels in BMSCs. These results support the notion that H_2_O_2_ induces oxidative stress in rat BMSCs. Moreover, treatment with H_2_O_2_ also attenuated the differentiation medium‐induced AKT activation and downstream FoxO1, as well as MyoD1 and myogenin expression in BMSCs. Because both MyoD1 and myogenin are important transcription factors for the myogenic differentiation of BMSCs [17, 18], the significant decrease in the levels of MyoD1 and myogenin expression induced by H_2_O_2_ indicates that oxidative stress not only affects the survival of BMSCs, but also attenuates the myogenic differentiation of rat BMSCs. It is possible that aberrant oxidative stress may damage the mitochondria and promote the mitochondrion‐dependent apoptosis of BMSCs [19]. These, together with the inhibition of AKT activation, which is an important signaling for cell survival and proliferation, reduced the cell viability. In addition, the decrease in FoxO1, MyoD1 and myogenin expression induced by H_2_O_2_ supports the notion that oxidative stress inhibits the myogenic differentiation of BMSCs and contributes to the pathogenesis of musculoskeletal dysfunction. Hence these findings may provide new insights into the pathogenesis of musculoskeletal dysfunction‐related diseases.

We found that although treatment with rhTrx alone did not alter the viability of BMSCs, this treatment enhanced the differentiation condition‐mediated AKT activation and FoxO1, MyoD1 and myogenin expression in BMSCs during the myogenic differentiation *in vitro*. Thus, the PI3K/AKT/FoxO1 signaling and the expression of MyoD1 and myogenin are important for the myogenic differentiation of BMSCs. It is well known that MyoD1 and myogenin are critical for the differentiation of BMSCs into myocytes and can be incorporated into regenerative muscle fibers [20, 21, 22]. In addition, the PI3K/AKT/FoxO1 signaling can promote the osteogenic differentiation and other lineages of BMSCs [2, 23, 24, 25, 26]. To the best of our knowledge, this is the first report that rhTrx enhances the PI3K/AKT/FoxO1 signaling and myogenic differentiation of rat BMSCs.

More importantly, treatment with rhTrx significantly mitigated or abrogated the H_2_O_2_‐reduced cell viability, the H_2_O_2_‐induced cell cycle arrest, the oxidative stress and H_2_O_2_‐decreased AKT activation, and FoxO1, MyoD1 and myogenin expression in BMSCs. Furthermore, treatment with Wor inhibited the PI3K/AKT signaling and completely abrogated the therapeutic effects of rhTrx in H_2_O_2_‐exposed BMSCs. This further supports that rhTrx enhances AKT activation during the myogenic differentiation of BMSCs. Therefore, rhTrx may be valuable for intervention of oxidative stress‐related musculoskeletal diseases.

## Conclusions

**Fig. 6:**
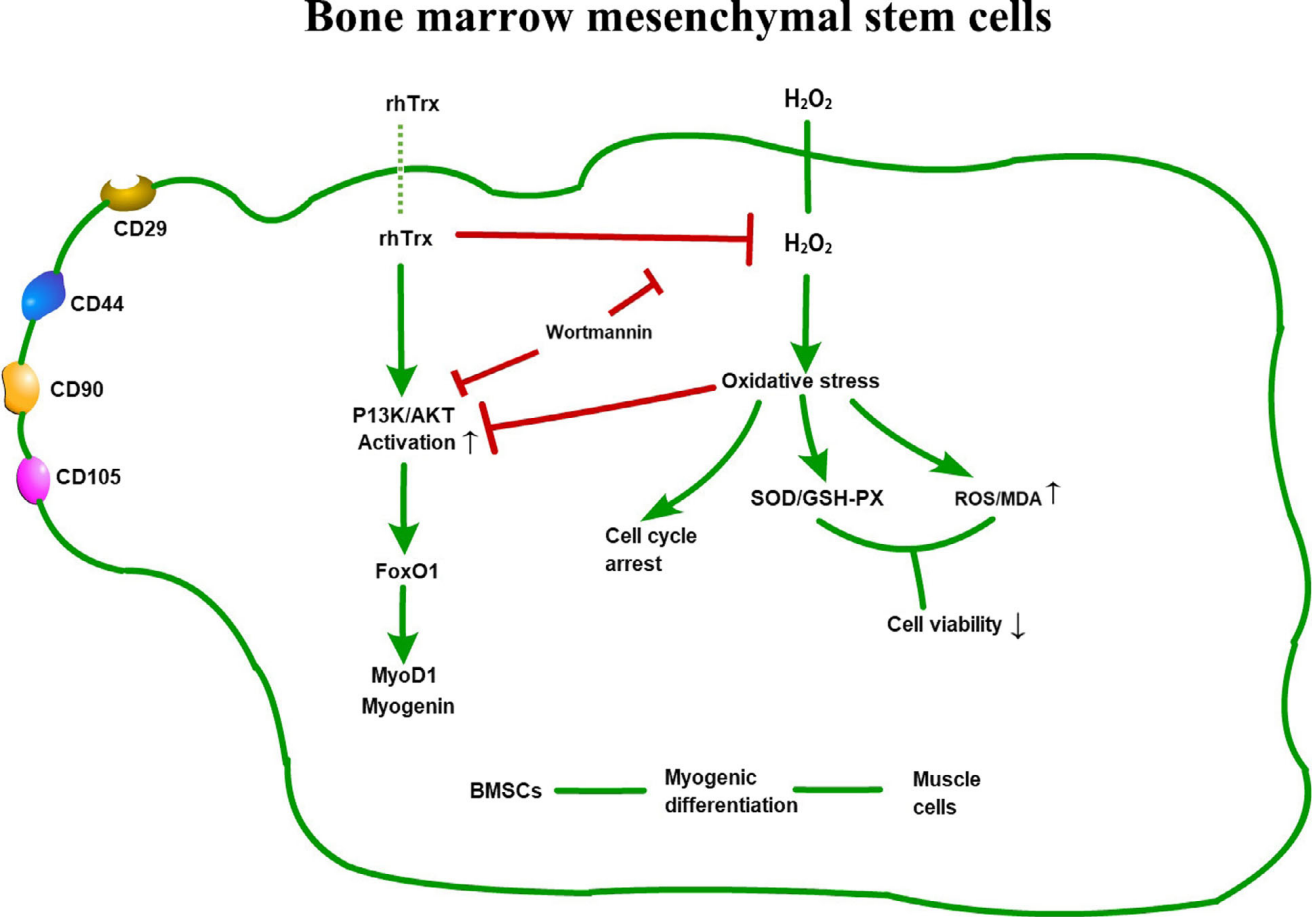
Illustration of the potential mechanisms underlying the action of Trx in mitigating the H_2_O_2_‐inhibited myogenic differentiation of rat BMSCs. H_2_O_2_ induced oxidative stress and reduced the viability of BMSCs by inducing cell cycle arrest and inhibiting the differentiation‐induced AKT activation and FoxO1, MyoD1 and myogenin expression, whereas treatment with rhTrx significantly mitigated or abrogated the H_2_O_2_‐induced oxidative stress and enhanced the PI3K/AKT/FoxO1 signaling to promote MyoD1 and myogenin expression, leading to myogenic differentiation of rat BMSCs.

These data indicate that H_2_O_2_ induces oxidative stress and reduces the viability of BMSCs by inducing cell cycle arrest and inhibiting the differentiation‐induced AKT activation and FoxO1, MyoD1 and myogenin expression. Treatment with rhTrx significantly mitigated or abrogated the H_2_O_2_‐induced oxidative stress to reduce ROS production, preserve antioxidant and promote cell cycling, and enhanced the PI3K/AKT/FoxO1 signaling to promote MyoD1 and myogenin expression, leading to myogenic differentiation of rat BMSCs (Fig. 6). Therefore, these findings may provide new insights into the pathogenesis of oxidative stress‐related musculoskeletal diseases and aid in the design of new therapies for the intervention of musculoskeletal diseases.

## Conflict of interest

The authors declare no conflict of interest.

## Author contributions

ML, XW and ZD conceived and designed the project. ML, XL and MW acquired the data. ML, HW, HD, LC, CZ and LG analyzed and interpreted the data. ML wrote the paper. CZ drafted Fig. 6.
